# Influence of Leaders' Psychological Capital on Their Followers: Multilevel Mediation Effect of Organizational Identification

**DOI:** 10.3389/fpsyg.2017.01776

**Published:** 2017-10-12

**Authors:** Qishan Chen, Zhonglin Wen, Yurou Kong, Jun Niu, Kit-Tai Hau

**Affiliations:** ^1^Guangdong Key Laboratory of Mental Health and Cognitive Science, Center for Studies of Psychological Application, School of Psychology, South China Normal University, Guangzhou, China; ^2^Department of Educational Psychology, Faculty of Education, The Chinese University of Hong Kong, Hong Kong, China

**Keywords:** psychological capital, organizational identification, work team, hierarchical linear model, multilevel mediation effect

## Abstract

We investigated the relationships between leaders' and their followers' psychological capital and organizational identification in a Chinese community. Participants included 423 followers on 34 work teams, each with its respective team leader. Hierarchical linear models (HLM) were used in the analyses to delineate the relationships among participants' demographic background (gender, age, marital status, and educational level), human capital, and tenure. The results revealed that leaders' psychological capital positively influenced their followers' psychological capital through the mediation effect of enhancing followers' organizational identification. The implications of these findings, the study's limitations, and directions for future research are discussed.

## Introduction

In recent years, research on positive organizational behavior (POB) has received increasing attention in the field of psychology and organizational science. As a branch of positive psychology, POB highlights and focuses on strengthening individuals' merits and virtues instead of screening away weaker individuals and focusing only on their weaknesses and mistakes. This perspective, which concentrates on strength, appears to be more beneficial in improving employees' job performance than the traditional approach, which creates keen competitiveness only among employees. In brief, POB argues that for both individual employees and the organization, it is important that the followers' positive psychological status (e.g., psychological capital) be recognized, cultivated and enhanced effectively so as to maintain their sustainable competitive advantages (Luthans, 2002; Luthans et al., 2006b; Wagner and Hollenbeck, 2014).

Considerable evidence of the importance of psychological capital has accumulated in the fields of organization science, human resource management, and other research areas (see reviews, Avey et al., 2011b; Youssef-Morgan, 2014). Most studies, however, have focused exclusively on the influence of followers' psychological capital on their output and productivity, such as work attitude and work behavior (Avey et al., 2011b). Little attention has been paid to understanding the interpersonal interaction of psychological capital within an organization or performing a systematic evaluation of the interactive mechanism between leaders and their followers' psychological capital. Researchers have generally used a single-level approach in their methodology and statistical analyses, treating all participants as independent individuals. A more appropriate analytical approach is to treat participants as groups of followers in relation to team leaders in a multilevel manner.

Recently, several studies have used hierarchical linear modeling (HLM) in an attempt to investigate the interpersonal interaction of psychological capital from a multilevel perspective (Walumbwa et al., 2010b; Ren et al., 2013). These studies have shown that leaders' psychological capital influences their followers' psychological capital, which subsequently exerts a positive effect on their work behavior and job performance. On one hand, these studies extend the former single-level analysis to a multilevel approach in the organizational context. On the other hand, these studies are of great importance in enriching our knowledge of interpersonal interaction within an organization from the perspective of positive psychology. Related research makes speculations but has not yet formally and empirically examined how leaders' psychological capital might affect their followers' psychological capital.

Based on the literature review of previous research, the purpose of the present study was to explore how leaders' psychological capital might influence that of their followers under the framework of social identity theory. From the perspective of POB, this study further examined the mediating effect of followers' organizational identity in the multilevel model. Specifically, this study investigated the cross-level mediating role of followers' organizational identification in the relationship between leaders' psychological capital and their followers' psychological capital in the social identity theory framework.

### Psychological capital

POB focuses on positive and high-performance-related psychological resources that can be measured, cultivated and managed effectively. These constructs include self-efficacy, hope, optimism, subjective well-being, emotional intelligence and other work-related desirable traits (Luthans, 2002). Seligman (2002) suggested that these positive psychological factors could be integrated into the construct of capital, which greatly broadens the scope of related research. Accordingly, based on analyses of the distinctions among economic capital, human capital and social capital, Luthans et al. (2006a,b, 2015) proposed the concept of positive psychological capital, which reflects the importance of individual positive psychological characteristics.

Psychological capital thus represents an important positive psychological state of development that is characterized by four psychological resources: (1) self-efficacy—confidence in the ability to take on challenging tasks and to succeed, (2) optimism—positivity toward current and future success, (3) hope—perseverance to accomplish a goal, and (4) resilience—ability to sustain and to bounce back when confronted with adversity (Luthans et al., 2006b, 2015). In other words, psychological capital is defined as a construct that measures an individual's positive psychological capacities, such as self-efficacy, optimism, hope and resilience, which are extend beyond economic, human and social capital.

The antecedents and consequences of psychological capital have been extensively researched. A meta-analysis of 51 independent samples, representing a total of 12,567 employees, showed that psychological capital may effectively support followers' job satisfaction, organizational commitment, and psychological well-being and thus may reduce job stress and job burnout. In addition, it can improve followers' organizational citizenship behavior as well as their objective and subjective career success. Psychological capital also reduces followers' turnover and deviance (Avey et al., 2011b).

Recently, researchers have been interested in exploring the role that psychological capital plays in interpersonal interaction within an organization using a statistically more appropriate multilevel approach. This approach is methodologically stronger than previous individual-level analyses on the antecedents and consequences of psychological capital. Recent evidence has suggested that leaders' psychological capital is positively related to their followers' performance as it enhances followers' psychological capital. In a sense, psychological capital is the mediator in the process. Moreover, service climate functions as a moderator that affects the relationship between followers' psychological capital and performance (Walumbwa et al., 2010a).

In the Chinese cultural context, Ren and his colleagues' research showed that leaders' psychological capital has a positive influence on followers' organizational citizenship behavior, with followers' psychological capital as a mediator (Ren et al., 2013). They adopted a multilevel approach in exploring the interaction between leaders' psychological capital and followers' work behavior. The more advanced and more appropriate multilevel approach is more ecologically valid than the individual-level approach adopted in previous studies. It also avoids ecological fallacy in organizational psychological studies. These studies, however, have not examined the reasons why followers' psychological capital functions as a multilevel mediator. To address this issue, social identity theory provides a strong and competitive explanation in that leaders' psychological capital is hypothesized to have both direct and indirect effects on their followers' psychological capital, with the followers' organizational identification as the mediator.

The effects of psychological capital have been reaffirmed in the Chinese cultural context. Studies have shown that self-efficacy, hope, optimism, resilience and other positive psychological attributes of Chinese followers are positively related to their job performance. Moreover, the positive relationship between psychological capital and job performance appears to be distinct across different domains of psychological attributes (Luthans et al., 2005). Congruent with Western findings, Zhong (2007) noted that psychological capital among Chinese team leaders exerts a positive influence on followers' job performance, organizational commitment and organizational citizenship behavior. These effects are generally larger than those of hope, optimism and resilience. These research findings indicate that the relationship between psychological capital and followers' work attitude and behavior in the Chinese cultural context is similar to that in the Western cultural context. In addition, the measured structure of psychological capital of studies in the Chinese cultural context is also consistent with the original scale reported in the Western countries (Shen et al., 2014). These findings indicate that psychological capital could be regarded as a higher-order factor of self-efficacy, optimism, hope, resilience and other positive psychological states.

### From leaders' psychological capital to followers' psychological capital

A work team consists of team members who hopefully have the same commitment and responsibility to achieve common team goals or tasks. Work teams are embedded in the larger organizational structure, and the team itself enacts a context for team members. Organizations, teams, and individuals are bound in a multilevel system and structure. In brief, individuals are nested in teams, and teams are linked to and nested in a larger organization. Though work teams operate in an organizational context, information communication and interpersonal interaction flow between the team leaders and followers and among followers (Sundstrom et al., 1990; Kozlowski and Bell, 2003; Mathieu et al., 2014; Gilson et al., 2015).

Psychological capital is conceptualized as a state-like characteristic that is susceptible to development and other interactive effects. In other words, psychological capital in the leader-follower relationship and that in the follower-follower relationship could easily impact one another through interpersonal interactions (Sy et al., 2005; Story et al., 2013).

Team leaders not only instruct and supervise their followers but also offer them information and resource in daily work. Followers obey leaders' orders and accomplish assigned tasks. During the process, leaders' psychological state may influence followers' work attitude and behavior formally or informally. Such an influence varies across different leaders. Leaders with rich psychological capital tend to be more hopeful, more motivated to succeed, and more likely to set up more challenging goals than leaders who lack the necessary psychological capital. Stronger leaders are more active in exploring solutions to overcome obstacles and are more willing to make an effort to succeed (Peterson et al., 2009; Avey et al., 2011a). At the same time, it is much easier for them to recover from adversity and failure. In other words, they have more positive expectations toward the work environment, resulting in a more positive attitude and excellent job performance. Such leader characteristics and behavior will positively influence their followers and will make them experience more positive psychological capacity and emotions. Subsequently, this will affect workers' work attitude and work behavior through improving their psychological capital (Bono and Ilies, 2006; Dinh et al., 2014). Team leaders lacking such a positive psychological capacity of confidence, optimism, hope and resilience will not be able to inspire or encourage their team workers.

Leaders are always regarded as role models for their followers, who usually imitate their leaders. In such a way, leaders become an influential source of information for their followers and thus have a great impact on the followers' attitude and behavior. If leaders display high levels of psychological capital, their followers will more readily pursue positive outcomes. These followers will therefore be more likely to develop positive and optimistic job expectations to maintain stronger motivation for success (Yammarino et al., 2008; Rego et al., 2012). Their expectations and motivation may help them build positive psychological capacities such as confidence, optimism, and resilience.

In this study, Hypothesis 1 is that leaders' psychological capital positively affects their followers' psychological capital.

### Leaders' psychological capital and followers' organizational identity

Leaders' psychological capital not only directly improves their followers' psychological capital but also affects it through enhancing followers' organizational identification. Individuals' social identity is a part of their self-concept, which derives from their perceived membership in a relevant social group (Ashforth and Mael, 1989). As a special form of social identity, team identification and organizational identification will lead to activities that are congruent with this identity and support the larger organization. These types of identification will also lead to outcomes that traditionally are associated with group formation, which reinforce the antecedents of identification (Hogg and Terry, 2000, 2014).

In the process of interpersonal interaction, followers tend to define their own working self-concept based on shared team values, which, through organizational socialization and leader-member exchange, helps build the person-organizational fit. In this way, individual socialization takes place gradually (Brown, 2000; Hogg and Terry, 2000, 2014; He and Brown, 2013; Haslam et al., 2014). As managers of work teams, leaders shoulder the responsibility to lead their teams to accomplish work tasks. Team leaders' psychological state and behavior shape the work context for followers and influence followers' work attitude and behavior through affecting their organizational identification (Zhu et al., 2012; Huettermann et al., 2014).

It has been demonstrated that leaders' positive psychological capital, such as their efficacy, optimism, resilience, and hope, may be immediate predictors of followers' organizational identification (Larson et al., 2013). In a study with 328 Chinese team members, Dou (2011) showed that leaders' psychological capacity (such as responsibility, hope, and resilience) can significantly predict followers' level of organizational identification. Leaders with a higher level of responsibility can improve followers' organizational identification by offering greater support to their followers and maintaining a higher level of work enthusiasm within teams. Leaders with a high level of hope have a better command of the working environment and provide different solutions for problems. Therefore, they can better fulfill their goals and gain greater trust from their followers (Lane and Chapman, 2011). Leaders with a higher level of resilience also possess more immunity to negative stressful events. They not only develop positive emotional control but also influence their followers in developing positive emotions. These behaviors will thus help the work team handle adversity and setbacks in a positive way and accordingly improve followers' organizational identification.

A number of studies have demonstrated that leaders give crucial clues in guiding followers in their interpretation of work events. Undoubtedly, leaders play an important role in the formation of team climate, and a healthy and harmonious team climate contributes to the improvement of organizational identification (Mayer et al., 2007; Walumbwa et al., 2010a; Gelfand et al., 2012). Leaders with high psychological capital can facilitate positive and efficient interpersonal interactions, effective solutions for misunderstandings and conflicts, and a healthy work climate, which greatly benefit followers' organizational identification. On the contrary, leaders without adequate psychological capital will more likely suppress followers' organizational identification.

In summary, leaders' psychological capital plays an indispensable role for their followers. Leaders can help followers interpret the interpersonal and social background at work. They also facilitate followers' ability to successfully integrate the shared psychological characteristics into the team. We therefore propose, in Hypothesis 2, that leaders' psychological capital positively affects their followers' organizational identification.

### Mediation of followers' organizational identification

Team identification, or organizational identification, is conceptualized as consistency in perception between an individual and a team or organization. It represents consistency in work values between an individual and a team, and it reflects followers' emotional unification in domains such as the sense of belonging, pride, and loyalty (Hogg and Terry, 2000; He and Brown, 2013; Haslam et al., 2014). Without a strong sense of identity, team members may easily develop negative psychological state, resulting in lack of work enthusiasm and inefficient output in job performance. The impact of an individual factor, especially individual organizational identification, therefore must not be neglected in exploring the extent to which leaders' psychological capital may influence followers' psychological capital.

Leaders are the organization members responsible for creating and maintaining work conditions for employees to achieve the team goals. Consequently, employees' social exchange within a team largely takes place through leaders (Hekman et al., 2009; Ashforth et al., 2013). Individual members create their identity through their relationships with the organization. When the relationships are strong enough to boost their self-esteem and make them treasure such relationships, they are willing to make more contributions to the organization and to improve their performance and the organizational image. At the beginning stage, employees and the organization are linked through their respective labor contracts. Only when employees psychologically consider themselves part of the organization will they develop more positive psychological states and work behavior.

Organization identification can be predicted by employees' perceptual and environmental variables, such as their perceived organizational reputation, their need for organizational identification, and their psychological capital, as well as organizational and supervisor support (Larson and Luthans, 2006; Ashforth et al., 2013; Haslam et al., 2014). Researchers have also revealed that organizational identification can predict employees' positive work attitude and behavior. The higher the level of employees' identification, the more likely they are to choose to solve problems from the organizational point of view and to develop behavior that is beneficial for the organization. Moreover, individuals' organizational identification may lead to depersonalization and make them have a sense of common fate with the organization, showing more cooperation and organizational behavior. In this way, employees are inclined to work harder, take initiative to assist their leaders with solutions for problems and seem to be more satisfied with their own work (Vora and Kostova, 2007; Hekman et al., 2009; He and Brown, 2013). In other words, organizational identification functions as a mediator between antecedent variables and work attitude or behavior.

Team identification is seen as a process in which members define themselves, subordinate themselves to the team, and build a psychological bond with the respective organization. We therefore speculate that leaders with high levels of psychological capital will be more successful in forming an honest and harmonious work team. The team will also have stronger mutual trust and an elevated team identification and sense of belonging. The more psychological capital employees perceive, the more likely they are to commit to the organization and, hence, the stronger the development of their efficacy, optimism, hope and resilience. We therefore propose Hypothesis 3 that followers' organizational identification serves as a mediator between leaders' psychological capital and their followers' psychological capital.

**Figure 1:**
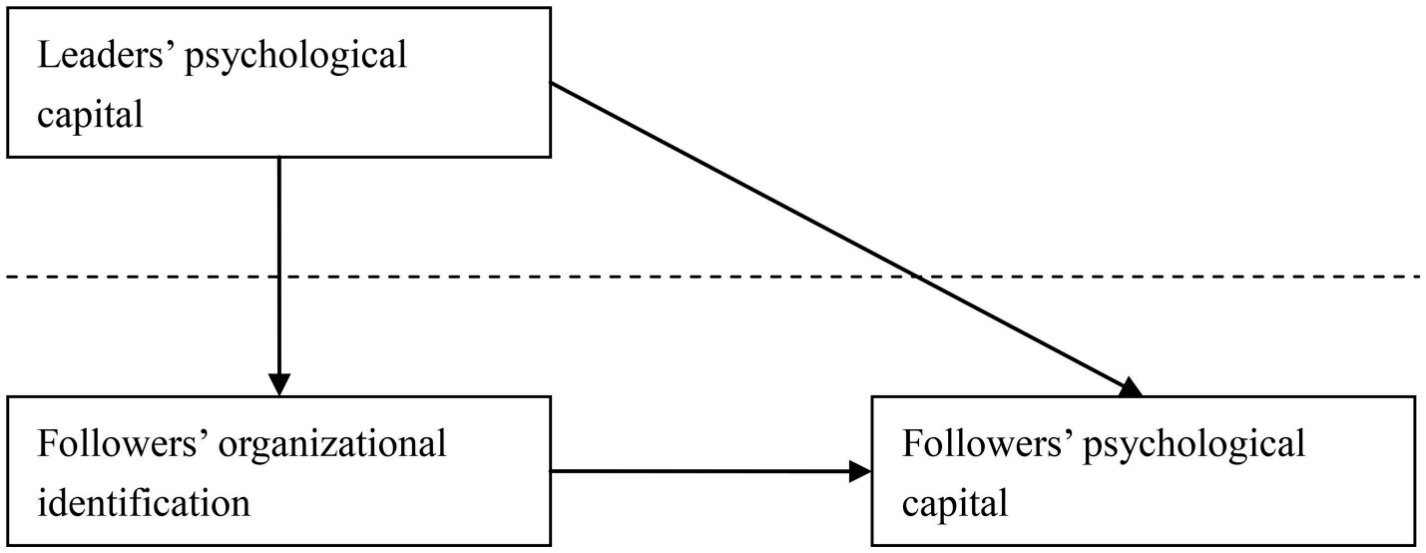
Hypothesized model.

To sum up, the hypothesized model is depicted in Figure 1.

## Materials and methods

### Participants

Participants in this study were work teams from three financial companies in a large city in southern China, recruited through a convenient sampling method. To minimize the impact of irrelevant factors, such as job characteristics and organizational culture, all companies were chosen from the same industry. The companies were similar in their organizational forms, work tasks, work patterns and performance assessment criteria. A total of 550 questionnaires were distributed, and 457 valid questionnaires were collected, for an 83.1% participation rate. Specifically, there were 34 work teams, with an average of around 13 members (ranging from 4 to 23) on each team. All members had worked with their direct supervisors for at least 1 year, and the average was 2.77 years.

Among the participants, 55.8% were males, and 44.2% were females; 58.5% of them were married and 41.4% were unmarried. In terms of age, 21.0% were between 18 and 25 years old, 33.0% between 26 and 33, 23.9% between 31 and 35, 13.3% between 36 and 40, 6.1% between 41 and 45, 2.0% between 46 and 50, and 0.7% over 50. The education background of the participants varied from middle school education to master's or above; 0.7% had a middle school education or below, 4.8% high school, vocational high school, technical school or technical secondary school, 19.5% junior college, 56.7% undergraduate education, and 18.4% master's education or above.

In terms of their working status, 67% participants were ordinary staff, 18.6% first-line managers, 12.0% middle managers and 2.4% senior managers. For monthly salaries, 10.9% participants earned 3,000 Chinese Yuan (CNY) or below (1 US$ is about 6.7 Chinese Yuan), 24.3% between 3,001 and 6,000 CNY, 29.1% between 6,001 and 9,000 CNY, 15.8% between 9,100 and 12,000 CNY, 10.7% between 12,001 and 15,000 CNY, and 9.2% between 15,001 and 18,000 CNY. The participants had an average length of service of 16.39 years, with a standard deviation of 10.95 years.

The present study has been approved by the Research Ethics Committee of the respective university. All participants provided their written consent before completing the questionnaires. The data were collected and analyzed anonymously.

### Variables measured

#### Psychological capital

We adopted the Chinese version of the PsyCap Questionnaire (PCQ) by Luthans et al. (2007) to measure participants' psychological capital. The Chinese version of the PCQ has been widely used and has been demonstrated to have adequate structural validity in multiple samples (Zhang et al., 2010; Ren et al., 2013; Shen et al., 2014). This 24-item Likert-type (1 = strongly disagree, 2 = disagree, 3 = somewhat disagree, 4 = somewhat agree, 5 = agree, 6 = strongly agree) scale contained four dimensions—efficacy, optimism, hope and resilience—with 6 items for each dimension. The internal consistency Cronbach's alphas of leaders' psychological capital in efficacy, hope, optimism, and resilience were 0.90, 0.83, 0.71, and 0.72, respectively; those of the followers' psychological capital were 0.87, 0.80, 0.76, and 0.73 respectively.

#### Team identification

We adopted the Mael scale, a 6-item scale, to measure the individual degree of identity on the work team. This one-dimensional scale was developed by Mael and his colleagues (Mael and Ashforth, 1992; Mael and Tetrick, 1992) and has been considered as the top choice among organizational identification measures due to its brevity and extensively demonstrated high validity and reliability. Cronbach's alpha coefficient of this 6-point scale measure was 0.88.

### Measurements and data analyses

We sent the self-report questionnaires to the team leaders and their followers separately. The questionnaire for the leaders was the psychological capital scale, while the scales for the followers consisted of both the psychological capital scale and the team identification scale. To relieve participants' concerns about privacy, all questionnaires and subject participation fees were enclosed in the envelopes distributed, which were sealed with the adhesive tape. Participants were instructed to seal the questionnaires with the envelopes after completion.

For the follower questionnaires, we adopted Harman's One-Factor Test to examine the common method variance (Malhotra et al., 2006). The goodness-of-fit indexes of the one-factor model and the two-factor model were, respectively, χ^2^_(27)_ = 562.01, RMSEA = 0.26, NNFI = 0.80, CFI = 0.85, SRMR = 0.12 and χ^2^_(26)_ = 142.22, RMSEA = 0.12, NNFI = 0.94, CFI = 0.95, SRMR = 0.063. The result that the two-factor solution was better suggested that the homologous coefficient of variance was possible but that its influence would be small. In addition, we conducted a test on cross-group measurement invariance using the multi-group confirmatory factor analysis on psychological capital.

The commercial statistical software SPSS (version 20.0) was used in our study to conduct descriptive statistics, correlation analyses, exploratory factor analyses and reliability analyses. We used LISREL 8.70 to conduct confirmatory factor analysis and HLM 6.02 to build the cross-level mediation-lower mediator model. The EM algorithm was adopted in imputing the missing data.

## Results

### Cross-group measurement invariance test

Since the psychological capital questionnaire was used for both the team leaders and the team members, it was necessary to ensure the similarity of the measurement structure in these two groups. Thus, we conducted a test on the cross-group measurement invariance by means of the multi-group confirmatory factor analysis methodology (Byrne, 1998). We imposed pattern invariance, factor loading invariance, indicator uniqueness variance invariance, factor variance invariance and factor covariance invariance sequentially to compare the restrained and unrestrained models. Through the model comparison, we were able to determine whether the model was cross-group measurement invariant. The results (see Table 1) indicate that Δχ^2^ (Δ*df*) of the models was not statistically significant and that the goodness-of-fit indexes did not show obvious deterioration. It was therefore concluded that the psychological capital instruments for team leaders and team members shared a similar factorial structure. That is, the psychological capital questionnaire in this study was applicable for the two different groups—team leaders and team members.

**Table 1:** Cross-group measurement invariance of psychological capital.

**Model**	**χ^2^**	***df***	**χ^2^/*df***	**Δχ^2^ (*df*)**	**Δχ^2^/*df***	**RMSEA**	**NNFI**	**CFI**	**SRMR**
M_0, L_	372.35	183	2.03	——	——	0.11	0.92	0.93	0.092
M_0, F_	703.34	183	3.84	——	——	0.10	0.95	0.95	0.062
M_1_	1,075.69	366	2.94	——	——	0.10	0.94	0.95	0.062
M_2_	1,096.87	387	2.83	21.18 (21)	1.01	0.10	0.94	0.95	0.068
M_3_	1,111.08	393	2.83	14.21 (6)	2.37	0.10	0.94	0.95	0.065
M_4_	1,140.32	414	2.75	29.24 (21)	1.39	0.10	0.95	0.95	0.065
M_5_	1,183.69	435	2.72	43.37 (21)	2.07	0.10	0.95	0.95	0.066

### Descriptive statistics

The results of the descriptive statistics are shown in Table 2. A few correlations are noted: (i) followers' psychological capital was positively correlated with followers' age, (ii) followers' team identification was positively correlated with followers' years of education, and (iii) followers' psychological capital was positively correlated with team identification. Male team followers' identification was also found to be significantly lower than that of female followers.

**Table 2:** Descriptive statistics.

**Variables**	***M***	***SD***	**1**	**2**	**3**	**4**	**5**	**6**	**7**
Leader psychological capital	4.22	0.46	–						
Follower sex^a^	0.77	0.42	–	–					
Follower age	34.15	9.42	–	0.153^**^	–				
Follower education years	13.41	1.78	–	−0.267^**^	−0.218^**^	–			
Follower work experience	16.39	10.95	–	0.146^**^	0.863^**^	−0.300^**^	–		
Follower psychological capital	4.00	0.50	–	−0.094	0.173^**^	0.033	0.092	–	
Follower team identification	4.18	0.65	–	−0.137^*^	0.069	0.153^**^	0.047	0.581^**^	–

** *p < 0.01*,

* *p < 0.05*.

a *0 = female, 1 = male (the mean value of sex represents the proportion of male followers in the sample). The leader psychological capital reflects group-level (work team) data*.

As seen from the results (Table 2), demographic variables (e.g., sex, age) and human capital (e.g., education, work experience) were related to followers' psychological capital and team identification. Thus, these variables were controlled for in our subsequent data analyses. We conducted regression analyses on the above independent variables and the dependent variables, such as team identification and psychological capital. We analyzed the residual errors of the dependent variables after controlling for the respective variables to reduce possible spurious effects on the results.

### Influence of leaders' psychological capital on followers' psychological capital: multilevel mediation effect of followers' team identification

Team leaders' psychological capital was a group-level variable, while followers' psychological capital and team identification were individual-level variables. We adopted a multilevel regression model to test the multilevel mediation effect. The model of this study was a cross-level mediation model, i.e., Model 2-1-1. We first centralized the Level 1 variables based on the group means. At the same time, we put such group means in the Level 2 intercept equation, which helped to separate the between-group mediation effect from the intra-class one, thus resulting in a more interpretable estimation of the multilevel mediation effect.

We began our analyses with a null model M_1_ to disintegrate the variance of dependent variables into two parts—intra-class variance, caused by individual differences, and between-group variance, due to group differences. We examined the percentage of between-group variance in dependent variables variance, i.e., *ICC*(1) (intra-class correlation coefficient). If *ICC*(1) was large enough, such as reaching 0.059, it meant that between-group differences existed and could not be neglected. In that case, the multilevel analysis using the HLM was well justified (Raudenbush and Bryk, 2002).

In our research, *ICC*(1) = 0.127/(0.127 + 0.121) = 0.512. The result showed that the factor at the group level accounted for 51.2% of the total variance in terms of followers' psychological capital and that in the individual level accounted for 48.8% of the total variance. Such large influences from different levels on followers' psychological capital indicated the necessity to adopt the HLM approach. The results revealed that the between-group variance was significant (τ_00_ = 0.127, χ^2^ = 481.154, *p* < 0.001); that is, there was a significant unexplained variance at the group level, and thus, the predictor variables were justified.

We followed a three-step procedure to test the cross-level mediation effect (see Table 3).

**Table 3:** Cross-level mediation effect of followers' team identification.

**Model**			**Estimated parameter**			
	**γ_00_**	**γ_01_**	**γ_02_**	**γ_10_**	**σ^2^**	**τ_00_**
M_1_: Null Model	3.99^***^				0.12	0.13^***^
L1: Fpsycap*_*ij*_* = β_0j_+ *r_*ij*_*						
L2: β_0j_ = γ_00_ + μ_0*j*_						
M_2_: Lpsycap → Fpsycap	1.64^***^	0.55^***^			0.12	0.08^**^
L1: Fpsycap_*ij*_ = β_0*j*_+ *r*_*ij*_						
L2: β_0j_ = γ_00_ + γ01^c^ (Lpsycap) + μ_0*j*_						
M_3_: Lpsycap → TI	1.50^***^	0.63^***^			0.22	0.12^*^
L1: TI*_*ij*_* = β_0j_+ *r_*ij*_*						
L2: β_0j_ = γ_00_ + γ01^a^ (Lpsycap) + μ_0*j*_						
M_4_: Lpsycap, TI → Fpsycap	1.39^***^	0.45^**^	0.17	0.24^***^	0.11	0.06^**^
L1: Fpsycap*_*ij*_* = β_0*j*_+ β_1*j*_ (TI) *+ r_*ij*_*						
L2: β_0j_ = γ_00_ + γ01^c′^ (Lpsycap) + γ_02_ (MTI) + μ_0*j*_						
β_1j_ = γ10^b^						

(1)

* *p < 0.05*,

** *p < 0.01*,

*** *p < 0.001; (2) σ^2^ represents Level 1 residual error, τ_00_ represents residual error of intercept, i.e., μ_0j_; (3) TI, Team Identification; Lpsycap, Leader psychological capital; Fpsycap, Follower psychological capital; MTI, Mean Team Identification (based on follower team identification at group level); (4) Level 1 variables centralized with mean value*.

In step 1, we built M2 to examine the direct effect (*c*) of the Level 2 independent variable *X*_*j*_ on the Level 1 dependent variable *Y*_*ij*_ to test the effect of team leaders' psychological capital on followers' psychological capital. The regression coefficient was statistically significant (γ01^c^ = 0.554, *t* = 5.291, *p* < 0.001), and leaders' psychological capital had a positive influence on followers' psychological capital. After adding leaders' psychological capital, the between-group variance of followers' psychological capital changed from 0.127 (null model) to 0.065 (M2), with an obvious decrease in the random effect. In brief, these results provided support for H1, i.e., that leaders' psychological capital has a positive effect on followers' psychological capital.

In Step 2, we built M_3_ to test the direct effect (*a)* of the Level 2 independent variable *X*_j_ on the Level 1 mediation variable *M*_ij;_ to test the direct effect of leaders' psychological capital on followers' team identification. The regression coefficient was also statistically significant (γ01^a^ = 0.63, *t* = 4.50, *p* < 0.001). Thus, H2 was supported, reaffirming that leaders' psychological capital had a positive effect on followers' team identification.

In Step 3, M4 was built to test the effects (*c'* and *b*) of the Level 2 independent variable (leaders' psychological capital) and the Level 1 mediation variable (followers' team identification). The results revealed that team identification had a positive effect on followers' psychological capital (γ10^b^ = 0.24, *t* = 6.68, *p* < 0.001). With the mediator in the model, as expected, the influence of leaders' psychological capital on followers' psychological capital dropped, but it remained statistically significant (γ01^c′^ = 0.45, *t* = 3.36, *p* < 0.01). In sum, team identification functioned as a partial mediation variable in the effect of leaders' psychological capital on followers' psychological capital, thus supporting H3.

## Discussions

### Mechanism of leaders' psychological capital effects on followers' psychological capital

The psychological capital of employees can effectively improve their positive work attitude and work performance and can be regarded as an important resource for individuals and work teams to attain sustainable competitive advantages (Luthans et al., 2006b, 2015; Avey et al., 2011b). The present study showed that leaders' psychological capital exerted a positive influence on followers' psychological capital and that this influence was mediated by followers' team identification. In other words, leaders' psychological capital was positively related to followers' positive psychological capital by providing followers with more positive team identification.

In line with what has been suggested in previous theoretical work (Walumbwa et al., 2010b; Ren et al., 2013), we found that leaders' psychological capital influenced followers' psychological capital in a direct way. Leaders' psychological capital likely exerts a positive influence on followers' psychological capital through teamwork and the interpersonal interaction between leaders and followers within the same work teams.

In a business organization, the exchanges between individuals and the organization take place through different channels. First, there are material exchanges, such as salary and payment. Second, there are psychological social exchanges, such as support, trust, self-esteem, and prestige. Such psychological exchanges help build a sense of obligation, reciprocity, and reliance with the organization, which material exchanges could never achieve (Settoon et al., 1996; Cropanzano and Mitchell, 2005). Based on the norm of reciprocity (Wu et al., 2006; Shen et al., 2011), when both the material and psychological needs of followers are satisfied by the organization, followers tend to show more desirable work attitudes and work behaviors. The more positive and beneficial behavior organizations show toward their followers, the higher the quality of the exchange relationship between followers and organization that will be built. Leaders equipped with adequate psychological capital, therefore, will tend to provide more positive resources for their followers. These leaders will have more positive social exchange with their followers, eventually enhancing their followers' psychological capital (Walumbwa et al., 2010b). Psychological capital thus could be regarded as a product of social exchange between individuals and their organizations.

The affective events theory argues that leaders' psychological capital can be considered an environmental variable perceived by the followers to influence their work attitude and behavior (Weiss and Cropanzano, 1996; Carlson et al., 2011). Positive psychological states such as confidence, optimism, hope, and resilience could inspire followers and make it easier for leaders to communicate with their followers, to reduce conflict and friction in the workplace, and eventually to inspire followers to cooperate better and work more efficiently. In contrast, negative psychological states or emotions could impair the harmony and unity of followers (Barsade, 2002; Walter and Bruch, 2008; Barsade and Gibson, 2012). That is, leaders with rich psychological capital play an important role in fostering an honest and harmonious relationship with mutual trust on their work teams, and they enhance their followers' sense of identity and belonging. This eventually stimulates the positive psychological capital of followers (Yammarino et al., 2008; Walumbwa et al., 2011).

Regarding Hypothesis 2, leaders' psychological capital was found to be positively related to followers' organizational identification; thus, the hypothesis was supported. We argued that this could be explained by the basic characteristics of organizational identification.

Social identity theory proposes that everyone has the need to attain a positive social identity. People also hope to belong to a positively evaluated group (Brown, 2000; Hogg and Terry, 2000). As a special form of social identification, organizational identification is conceptualized as a part of self-concept derived from the understanding of the social groups to which an individual belongs (Tajfel, 2010). Thus, people tend to establish their own and others' social identity according to the social categories to which they belong. However, in most cases, being in a specific social category does not increase or reduce individuals' social identification. Only through social comparison does the value of the special social and team membership stand out. When their social groups are comparatively advantaged, members tend to attain higher self-esteem and a stronger sense of belonging and identification with their groups and teams (Zhang and Zuo, 2006).

Leaders who are characterized by efficiency, positivity, and resilience have the capacities to stimulate self-motivation in work and to mobilize resources effectively. In this way, their followers receive more support from their superiors, develop a stronger sense of pride and experience membership of the work team as special. Adequate psychological capital can allow team leaders to be equipped with integrity, making the leaders elicit good faith that they will act in accordance with their own words in carrying out their leadership. In this way, leaders with integrity are in favor of not only achieving team goals but also persisting in principle to the hilt. The faithful behavior of leaders in the long run is perceived by followers and thus helps improve their sense of identity (Harvey et al., 2006; Gardner et al., 2011). Additionally, researchers (Walumbwa et al., 2010b) have shown that leaders with more confidence, hope, resilience, and optimism are inclined to work harder and persist longer. They have more positive expectations for the environment and are more capable of quick recovery from adversity and failure. In this context, followers' sense of belonging toward the organization will be improved accordingly. Thus, work teams with leaders who have rich psychological capital tend to show better team performance, a more promising future and an advantageous status in social comparison. Team members whose self-esteem is high acquire a sense of superiority over other groups and in turn develop a stronger sense of team identification. Therefore, leaders' psychological capital is positively related to followers' organizational identification.

Hypothesis 3 postulated that organizational identification is a cross-level mediator between leaders' psychological capital and followers' psychological capital. Our study supported this hypothesis and argued that this mediating effect of organizational identification again reaffirmed its important functions.

Organizational identification comes from one's understanding of the social groups to which they belong. It highlights the importance of the emotional value of membership in specific groups. The self-categorization theory argues that we are inclined to categorize things automatically and incorporate ourselves into such categories, endowing ourselves with group characteristics in order to attain self-categorization. Such self-categorization enhances team members' hope at work. For instance, they will pursue goals with perseverance and adjust their approaches if necessary to achieve success (Turner and Reynolds, 2011).

Self-concept mainly comes from the identification of team members with their organization (Hogg and Terry, 2000). The social comparison contributes to individuals' self-evaluation and reduces the uncertainty of self-concept. From such a social comparison, followers have a better understanding of the organization's status and value. They realize that people can also gain their status and value through their membership, and this will result in higher self-efficacy. In other words, when faced with challenging work, these followers tend to have greater confidence and will exert the necessary effort to succeed.

To maintain advantageous membership, individuals within their respective organizations are inclined to maintain consistency with other team members, who also embrace and internalize the organization characteristics. When an organization is advantaged in social comparison, members attain a higher status and more value. Thus, members tend to be more optimistic and have positive attributions regarding their current and future success.

In organizations that have leaders with high psychological capital, followers who receive more positive evaluations and experience higher self-value are more likely to achieve greater success and match their personal goals with the organizational goals. Even confronted with adversities and problems, these people will recover quickly, surpass the difficulties and persevere to achieve success, showing greater resilience in the process.

In conclusion, individuals in organizations led by leaders with high psychological capital tend to define themselves as members of their respective organizations and see the team objectives, interests and specifications as their own. In addition, they will become more positive, optimistic, and full of hope, trying hard to maintain harmony with their organizations. Therefore, leaders' psychological capital stimulates followers' positive psychological capital through the mediating effects of followers' organizational identification.

### Implications

The method and results of the present study have two theoretical implications for other research on organizational behavior and psychological capital. We adopted HLM to explore the relationships between cross-level variables in the context of the organization, which enhances the ecological validity and the relationships between team leaders and their followers.

The attitudes and behaviors of people in the organization are influenced by many factors in the multilevel model. The multilevel approach enables the understanding of the interactions at the individual, group, organization, and society levels. This statistical model enables organizational psychologists to focus appropriately on the roles and explanatory power of the multilevel variables in related theories (Klein and Kozlowski, 2000). In this study, members were nested in the organization. Members' psychological capital and organizational identification belong to the first level of the multilevel data structure, and leaders' psychological capital belongs to the second level of the data structure.

If we ignored the multilevel structure of the data and used a traditional linear model (such as variance analysis and regression analysis), it would not only weaken the theoretical explanatory power but also reduce the external validity of the results (Zhang, 2010). Therefore, we used HLM in the data analyses and model construction to test the direct effect of the Level 2 independent variables (i.e., leaders' psychological capital) on the Level 1 dependent variable (i.e., members' psychological capital). We also examine the multilevel mediating effect of the variables (i.e., members' organization identification).

The results of the current study suggest that leaders' psychological capital not only has a direct influence on members' psychological capital but also has an indirect influence on members' psychological capital through the members' organizational identification. If all members come from one team, it is easy to understand that the leaders' psychological capital will have a direct influence on the members' psychological capital. However, if the members are nested in the team with their respective leaders, we will still find a positive relationship between the variables. These findings show the appropriate influence of the group-level construct (leaders' psychological capital) on the individual-level construct (members' psychological capital) without committing the ecological fallacy problem.

In addition, we should differentiate the variances from two sources in the organizational identification. One is the individual differences between the members of the same team, and another is the group-level difference due to different leaders. Therefore, the influence of leaders' psychological capital on members' psychological capital through organizational identification will vary across teams. Our results show that even if participants are from different teams, the mediating effect of organizational identification is still statistically significant, indicating that this multilevel mediation effect has strong cross-situational consistency and cross-organizational consistency. It also shows that our conclusion has sufficient ecological validity and explanatory power.

We explored how leaders' psychology capital affects their followers' psychological capital under the framework of social identity theory. First, we found that leaders' psychological capital, a Level 2 variable, could directly influence followers' psychological capital, a Level 1 variable. Moreover, we found that the relationship between leaders' psychological capital and their followers' psychological capital was mediated by followers' organizational identification, another Level 1 variable. In other words, followers' organizational identification functioned as a cross-level partial mediator in the relationship between leaders' psychological capital and followers' psychological capital. As a psychological bond that connects individuals and their organizations, organizational and team identification play important roles in the interaction between leaders and their followers. Thus, our research not only explored how leaders' psychology capital may influence their followers' psychological capital but also examined how organizational identification might affect individuals' state of mind and their work performance.

Furthermore, our results have several practical implications for organizational management and team construction. First, psychological capital serves as a facilitator and catalyst for individuals' positive work attitude and behavior within an organization. At the same time, it is crucial for a work team or an organization to maintain efficient functioning and long-term development. We should therefore pay attention to cultivating and developing both leaders' and followers' psychological capital in the process of enterprise management. In addition, due to the positive influence of leaders' psychological capital on followers' psychological capital, we should focus in particular on the improvement of leaders' psychological capital.

Followers' organizational identification can positively and directly predict their psychological capital. Additionally, it functions as a cross-level mediator for the relationship between leaders' psychological capital and followers' psychological capital. We could therefore promote followers' organizational identification through various approaches and facilitate the exploitation and accumulation of followers' psychological capital. Leaders also have to be more cautious when exercising their leadership to increase followers' identification, which will ultimately lead to an improvement of their psychological capital.

### Limitations and suggestions for future research

The data for this study came from two independent questionnaires measuring team leaders and followers. Although we controlled for the possible influences of variables such as demographics, education background and work experience, this cross-sectional study still has unavoidable serious limitations in making claims of causation among the variables. Due to pre-existing differences between industries in their production mode, organizational culture, and employee welfare, and because participants were selected by the convenience sampling method, we should be extremely cautious in generalizing this study to other industries or organizations. In addition, it is important to note that this study was conducted with participants (leaders and followers) in a Chinese cultural context. Given the numerous differences between the Oriental and Western cultures and the distinct characteristics of interpersonal interaction within a culture, the results of this study should be cautiously applied in cross-cultural contexts.

In light of the above limitations in the current research, we offer several suggestions for future related research. First, more research should be conducted in a cross-cultural context to explore the interaction between leaders' and followers' psychological capital and the mediation effect of followers' organizational identification in different cultures. Second, to improve the external validity, we recommend more diversified samples from different enterprises in different industries. Finally, other than social identity theory, theories such as social learning, social exchange, and emotional contagion have been suggested by several researchers to interpret the interactions between leaders' and followers' psychological capital. Future work could examine these different hypotheses and compare their power in explaining or capturing the richness of the interactions between leaders and followers.

## Author contributions

QC contributed to developing the theoretical framework, data analysis, organization, and overall writing of the paper. ZW contributed to the editing and organization of the paper as well as the overall design. YK, JN, and KH contributed to the design, data analysis, and editing of the paper.

### Conflict of interest statement

The authors declare that the research was conducted in the absence of any commercial or financial relationships that could be construed as a potential conflict of interest.
